# EARLY AND LONG-TERM OUTCOME OF SURGICAL INTERVENTION IN CHILDREN WITH INFLAMMATORY BOWEL DISEASE

**DOI:** 10.1590/0102-672020200002e1518

**Published:** 2020-11-20

**Authors:** Farbod KHOSRAVI, Pardis ZIAEEFAR

**Affiliations:** 1 Shohada-e-Tajrish Hospital, Shahid Beheshti University of Medical Sciences, Tehran, Iran

**Keywords:** Children, Inflammatory bowel disease, Surgery, Crianças, Doença inflamatória intestinal, Cirurgia

## Abstract

**Background:**

Although children with inflammatory bowel disease (IBD), disease control is possible through medical procedures, but surgical intervention is indicated in some cases.

**Aim:**

To evaluated long-term surgical outcomes in children with IBD.

**Methods:**

This retrospective cohort study was done on 21 children suffering IBD with surgical indication admitted to a referral children hospital in Tehran in 2019. The baseline information was collected by reviewing the recorded files and children were followed-up to assess surgical outcome.

**Results:**

The rate of early complications after surgery was 47.6%; they included intestinal perforation in 4.8%, peritonitis in 4.8%, wound infection in 23.8%, pelvic abscesses in 14.3%, deep vein thrombosis in 4.8%, intestinal obstruction in 9.5%, pancreatitis in 9.5% and anal fissure in 4.8%. The mean duration of follow-up for patients was 6.79±4.24 years. The rate of delayed complications during follow up was 28.6%. Accordingly, long-term free-complication survival rate during 5-10 years after surgery was 92.3% and 56.4%, respectively. Among the early features, lack of prior drug treatment and bleeding as indication for surgery, were two predictors of long-term surgical complications.

**Conclusion:**

Standard surgery in the treatment of IBD in children with surgical indication is associated with favorable outcome, although short- and long-term surgical complications are also predictable.

## Graphical Abstract

[Fig f1a]

**Figure f1a:**
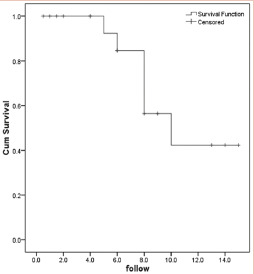
Long-term complication-free survival chart in children with IBD

## RESUMO

**Racional:**

Em crianças com doença inflamatória intestinal (DII) o controle da doença é possível através de procedimentos médicos; contudo, intervenção cirúrgica é necessária em alguns casos.

**Objetivo:**

Avaliar os resultados cirúrgicos em longo prazo em crianças com DII.

**Métodos:**

Este estudo é coorte retrospectiva realizado em 21 crianças com DII com indicação cirúrgica admitida em um hospital infantil de referência em Teerã, Iran, em 2019. As informações foram coletadas através da revisão dos arquivos e as crianças foram acompanhadas para avaliar o resultado cirúrgico.

**Resultados:**

A taxa de complicações precoces pós-operatória foi de 47,6%; incluíram elas perfuração intestinal em 4,8%, peritonite em 4,8%, infecção de ferida em 23,8%, abscessos pélvicos em 14,3%, trombose venosa profunda em 4,8%, obstrução intestinal em 9,5%, pancreatite em 9,5% e fissura anal em 4,8%. O tempo médio de seguimento dos pacientes foi de 6,79±4,24 anos. A taxa de complicações tardias durante o acompanhamento foi de 28,6%. Consequentemente, a sobrevida livre de complicações em longo prazo, durante 5 a 10 anos após o procedimento, foi de 92,3% e 56,4%, respectivamente. Entre as características iniciais, falta de tratamento medicamentoso prévio e sangramento como indicação para cirurgia, foram dois preditores de complicações cirúrgicas em longo prazo.

**Conclusão:**

O tratamento cirúrgico no tratamento da DII em crianças está associado a resultado favorável, embora complicações cirúrgicas de curto e longo prazo também sejam previsíveis.

## Graphical Abstract

[Fig f1as]

**Figure f1as:**
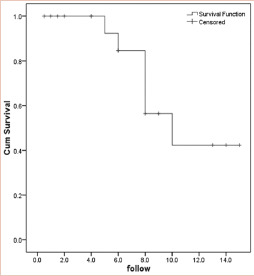
Sobrevida livre de complicações em longo prazo em crianças com DII

## INTRODUCTION

Inflammatory bowel disease (IBD) consists of two distinct but related conditions of chronic and recurrent inflammatory disorders in the gastrointestinal tract, including Crohn’s disease and ulcerative colitis. The first is characterized by patchy transmural inflammation with small bowel involvement as well as colon with different and varied clinical manifestations^3^. In contrast, ulcerative colitis is classically manifested by rectosigmoid mucosal inflammation^14^ ^,^ ^15^. About a quarter of IBD cases occur in childhood and adolescence^9^ ^,^ ^13^. In this respect, the incidence of Crohn’s disease in children is estimated at about 500.000 cases per year^2^. The average age of the disease in children is 12 years and only 5% of cases are under five^1^.

Treatment is mainly attempted to control the disease and relieve its symptoms through the use of medication and medical methods, but in some cases, such as exacerbation of pain, examination of the causes of rectal bleeding and anal pain, referral to the surgical center is necessary^5^ ^,^ ^11^. However, consideration should be given to the adverse effects and consequences of surgery, especially in the age group of children; as such complications in childhood can have a significant impact on their quality of life as well as their psychological aspects^6^ ^,^ ^16^.

The objective of this study was to evaluate surgical outcomes in children with IBD in order to consider the need for surgical management and management of surgical complication by evaluating outcomes in 1, 5, and 10 years after surgery. 

## METHODS

This study was a retrospective cohort. All children with IBD with surgical indication admitted to referral children hospital in Tehran in 2019 were included. Patients with incomplete data in the records, especially the lack of one-year follow-up information on surgical outcomes, were excluded. After designing the checklist, a complete overview of children with IBD with surgical indication was performed and information on demographic characteristics (gender and age), surgical indications, duration of medical and drug treatment, type of surgical procedure, complications following surgery and methods of improving and controlling these complications as well as the consequences of long-term after surgery in these patients were extracted and finalized. Researchers at all stages of their research were committed to the Helsinki Treaty, and the information of the participants was used without disclosing their identities. Individuals’ data were coded so that their names would not be used. All cases were monitored and approved by Shahid Beheshti University of Medical Sciences, Tehran, Iran.

### Statistical analysis

The results were presented as mean ± standard deviation (SD) for quantitative variables and were summarized by absolute frequencies and percentages for categorical variables. Normality of data was analyzed using the Kolmogorov-Smirnoff test. Categorical variables were compared using chi-square test or Fisher’s exact test when more than 20% of cells with expected count of less than five were observed. Quantitative variables were also compared with t test, Mann U, ANOVA or Kruskal-Wallis H tests. The survival was evaluated using the Kaplan-Mayer survival analysis. The multivariable regression modeling was used to determine the determinants of patients’ outcome. For the statistical analysis, the statistical software SPSS version 16.0 for windows (SPSS Inc., Chicago, IL) was used; p-values of 0.05 or less were considered statistically significant.

## RESULTS

Of the 22 patients enrolled, 21 underwent surgery and one improved with drug management and no surgical indication was done. Therefore, the statistical analysis of data was focused on 21 patients undergoing surgery. The mean age of the patients was 11.12±5.65 years ranged from 3-20 years. In terms of gender distribution, nine (42.9%) were male and 12 (57.1%) female. Mean time to treatment was 4.45±1.80 years. The most common indication for surgery was anemia in 100%, followed by intestinal bleeding in 33.3%, failure to thrive in 14.3%, recurrent defecations in 9.5% and severe abdominal colic in 14.3% (Table 1). 

**TABLE 1:** Baseline characteristics of patients

Mean age, year	11.12 ± 5.65
Male gender	9 (42.9)
Average time under medication, year	4.45±1.80
Surgery indications	
Anemia	21 (100)
Bleeding	7 (33.3)
Failure to thrive	3 (14.3)
Repeated defecation	2 (9.5)
Severe colic pains	3 (14.3)
History of liver transplantation	2 (9.5)
Type of surgery	
Coloproctectomy, ileoanostomy, GP, ileostomy	19 (90.5)
Colectomy, Hartmann, Ileostomy	2 (9.5)

In total 90.5% underwent standard total coloprotectomy with endorectal graft ileoanal anastomosis with ileal lobe ileostomy that was closed two years after surgery. In the remaining two patients (9.5%), the procedure was limited to total colectomy, Hartmann’s surgery and terminal ileostomy. In terms of early surgical complications, intestinal perforation occurred in 4.8%, peritonitis in 4.8%, wound infection in 23.8%, pelvic abscesses in 14.3%, deep vein thrombosis in 4.8%, intestinal obstruction in 9.5%, pancreatitis in 9.5% and anal fissure in 4.8%. Accordingly, the rate of early complications after surgery was 47.6% in total. Laparotomy was required in 23.8% to improve early surgical complications.

**FIGURE 1:**
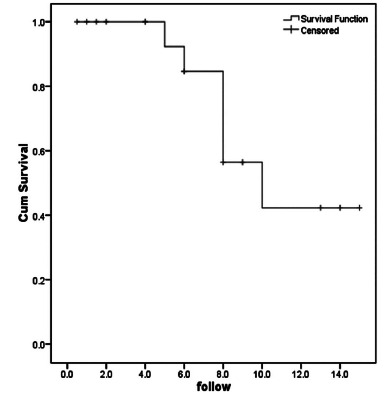
Long-term complication-free survival chart in children with IBD

The mean duration of follow-up for patients was 6.79±4.24 years, ranging from six months to 15 years. During this period, improvement in gastrointestinal function was reported in 100% of patients. However, in terms of long-term surgical complications, 23.8% had delayed fistulas and fecal incontinence was also reported in 4.8%. Also, the rate of delayed complications during follow up was 28.6%. Accordingly, long-term free-complication survival rate during 5 and 10 years after surgery was 92.3% and 56.4%, respectively (Figure 1).

Comparison between pre- and postoperative characteristics in the two groups with and without post-surgical complications (Table 2) showed significantly shorter average time under medication and higher rate of intestinal bleeding as the indication for surgery in the group with postoperative complications. 

Thus, among the early features, lack of prior drug treatment and bleeding as an indication for surgery were two predictors of long-term surgical complications. 

**TABLE 2:** Comparing baseline characteristics between the groups with and without complications

Item	With event	Without event	p
Mean age, year	9.16±4.66	11.90±5.95	0.329
Male gender	3 (50.0)	6 (40.0)	0.676
Average time under medication, year	3.25±1.75	4.93±1.63	0.049
Surgery indications			
Anemia	6 (100)	15 (100)	1.000
Bleeding	4 (66.7)	3 (20.0)	0.040
Failure to thrive	2 (33.3)	1 (6.7)	0.115
Repeated defecation	0 (0.0)	2 (13.3)	0.999
Severe colic pains	0 (0.0)	3 (20.0)	0.526
History of liver transplantation	0 (0.0)	2 (13.3)	0.999
Type of surgery			0.999
Coloproctectomy, ileoanostomy, ileostomy	6 (100)	13 (86.7)	
Colectomy, Hartmann, Ileostomy	0 (0.0)	2 (13.3)	

## DISCUSSION

Although in many patients, especially children with IBD, disease control is possible through medical procedures and is associated with acceptable therapeutic outcomes, but in some cases, particularly in the context of anemia, gastrointestinal bleeding or severe manifestations of inactivity, surgical interventions are indicated. In this regard, various techniques such as total coloprotectomy with endorectal graft ileoanal anastomosis with ileostomy loop are commonly used. So far, no comprehensive study has been published on the consequences of surgery in children with IBD in Iran, and this study was performed for this purpose. An overview of the results of our study showed that IBD was reported in children with surgical indication equally in both boys and girls, with a mean age of 11.12±5.65 years. In terms of indications for surgery, anemia was reported as the most important indication in all patients evaluated, whereas other indications included hemorrhage, failure to thrive, recurrent defecations, and severe colic, respectively. The dominant surgical technique included total coloproctectomy with endorectal graft ileoanal anastomosis with ileal loop anesthesia or total colectomy, Hartmann’s operation, and terminal ileostomy. In terms of early and in-hospital complications, the most common complications were wound infection in 23.8% of patients and pelvic abscesses in 14.3% of patients. Interestingly, in 23.8% of patients, laparotomy was indicated to control early surgical complications. In long-term follow-up, although remission in almost all patients undergoing surgical treatment was achieved, long-term surgical complications (including fistula or fecal incontinence) were reported in 28.6% of patients with two main predictors of not using primary drug therapy and intestinal hemorrhage as surgical indication. Finally, it was stated that the long-term complication-free survival within 5 and 10 years after surgery was estimated to be 92.3% and 56.4%, respectively, and the majority of delayed complications occurred after five years of surgery. In fact, it seems that IBD surgical treatment in children is fundamentally associated with a high success rate. However, short- and long-term complications are predictable in about a quarter of patients, which can be improved and controlled with appropriate measures. 

Different reports have been released regarding the prevalence of complications of IBD-related surgery in children, and even in some reports, the number is similar to that in colon cancer. In some studies, the complication rate after surgery was 18% and the need for reoperation was 7.3%^8^. Overall, the rate of recurrence rates among children under surgery was 50%, 73%, and 77% at 1, 5, and 10 years, respectively^4^. Therefore, it seems that our center has been largely successful in the surgical treatment and control of its complications and has been associated with complete improvement in patients, although short and long-term effects have also been of course controllable. 

Regarding the epidemiological aspects of IBD among children, our children appear to be within the prescribed gender, age, and clinical manifestations of other societies and reports. In international reports, 25% of IBD cases occur before the age of 20 years, while among children with IBD, 4% occur under the age of five and 18% under 10, and therefore most of the adolescent involvement will be in early youth^16^. In the present study, 13.6% of patients were under five years and 45.5% were under 10. In terms of clinical manifestations, the most prominent manifestations include anemia, developmental disorder, perianal diseases and some extraintestinal complications such as dermatological disorders, arthritis, osteopenia, autoimmune hepatitis, ophthalmic complications, nephrolithiasis, pancreatitis and thromboembolism. Our study also described anemia and developmental disorder at the top of the disease symptoms. The results of our study appear to be consistent with previous studies in terms of therapeutic outcomes of IBD. In a study by El-Baba et al^7^ in the United States, surgical indications included failure in medical treatment, complete or partial bowel obstruction, growth retardation, intestinal perforation, and abscess or fistula. In their research, in 47% of patients, complete remission was seen at one-year follow-up, but 22% had recurrence, with the majority of patients undergoing controlled medical treatment and two requiring reoperation. In 2012, in a study by Laituri et al^12^ from five patients who underwent surgery three had obstruction or stenosis and two had perforation. Knod et al.^11^ related a significant decrease in the frequency of defecation after surgery. 

Few studies have been performed to identify the predictors of surgical complications and, in our study, the history of medical treatment and bleeding as a surgical indication were identified as two predictors of surgical poor outcome. In a study by Dukleska et al.^6^ overweight and obesity along with the use of steroids in preoperative treatment increased the likelihood of surgical complications. Overall, considering the importance of predicting surgical complications, especially in the long-term, researches on the identification of postoperative complications are essential. 

## CONCLUSION

Standard surgery in the treatment of IBD in children with surgical indication is associated with favorable outcome, although short- and long-term complications are also predictable. Among the early features, lack of prior drug treatment and bleeding were two predictors of long-term surgical complications. Overall, long-term complication-free survival within 5 and 10 years after surgery was estimated to be 92.3% and 56.4%, respectively. 

## References

[1] Benchimol EI, Fortinsky KJ, Gozdyra P (2011). Epidemiology of pediatric inflammatory bowel disease a systematic
review of international trends. Inflamm BowelDis.

[2] Murch SH, Baldassano R, Buller H, Chin S, Griffiths AM, Hildebrand H, Jasinsky C, Kong T, Moore D, Orsi M (2004). Inflammatory bowel disease Working Group report of the second
World Congress of Pediatric Gastroenterology, Hepatology, and
Nutrition. J Pediatr Gastroenterol Nutr.

[3] Passos MAT, Chaves FC, Chaves-Junior N (2018). The importance of colonoscopy in inflammatory bowel
diseases. Arq Bras Cir Dig.

[4] Mamula P, Markowitz JE, Baldassano RN (2003). Inflammatory bowel disease in early childhood and adolescence:
special considerations. Gastroenterol Clin North Am.

[5] Kim SC, Ferry GD (2004). Inflammatory bowel diseases in pediatric and adolescent patients
clinical, therapeutic, and psychosocial considerations. Gastroenterology.

[6] Baldassano RN, Piccoli DA (1999). Inflammatory bowel disease in pediatric and adolescent
patients. Gastroenterol Clin North Am.

[7] Andres PG, Friedman LS (1999). Epidemiology and the natural course of inflammatory bowel
disease. Gastroenterol Clin North Am.

[8] Knod JL, Holder M, Cortez AR, Martinez-Leo B, Kern P, Saeed S, Warner B, Dickie B, Falcone RA, von Allmen D, Frischer JS (2016). Surgical outcomes, bowel habits and quality of life in young
patients after ileoanal anastomosis for ulcerative colitis. J Pediatr Surg.

[9] Dalal DH, Patton D, Wojcicki JM, Clark AL, Garnett EA, Heyman MB (2012). Quality of life in patients postcolectomy for pediatric-onset
ulcerative colitis. J Pediatr Gastroenterol Nutr.

[10] Pini-Prato A, Faticato M, Barabino A, Arrigo S, Gandullia P, Mazzola C (2015). Minimal invasive surgery for paediatric inflammatory bowel
disease personal experience and literature review. World J Gastroenterol.

[11] Dukleska K, Berman L, Aka AA, Vinocur CD, Teeple EA, Short-term outcomes in children undergoing restorative
proctocolectomy with ileal-pouch anal anastomosis (2018). J Pediatr. Surg.

[12] Kelley-Quon LI (2012). Postoperative complications and health care use in children
undergoing surgery for ulcerative colitis. J Pediatr Surg.

[13] Carrie A (2011). Laituri, MD, Jason D Fraser, MD, Carissa L. Garey, MD, et al.
Laparoscopic Ileocecectomy in Pediatric Patients with Crohn's
Disease. J Laparoendosc Adv Surg Tech A.

[14] Knod JL, Holder M, Cortez AR, Martinez-Leo B, Kern P, Saeed S, Warner B, Dickie B, Falcone RA, von Allmen D, Frischer JS (2016). Surgical outcomes, bowel habits and quality of life in young
patients after ileoanal anastomosis for ulcerative colitis. J Pediatr Surg.

[15] El-Baba M1, Lin CH, Klein M, Tolia V (1996). Outcome after surgical intervention in children with chronic
inflammatory bowel disease. Am Surg.

[16] Laituri CA, Fraser JD, Garey CL, Aguayo P, Sharp SW, Ostlie DJ, Holcomb GW, St Peter SD (2011). Laparoscopic ileocecectomy in pediatric patients with Crohn's
disease. J Laparoendosc Adv Surg Tech A.

